# Testing of Chemically Activated Cellulose Fibers as Adsorbents for Treatment of Arsenic Contaminated Water

**DOI:** 10.3390/ma14133731

**Published:** 2021-07-02

**Authors:** Mihaela Ciopec, Gabriela Biliuta, Adina Negrea, Narcis Duțeanu, Sergiu Coseri, Petru Negrea, Makarand Ghangrekar

**Affiliations:** 1 Faculty of Industrial Chemistry and Environmental Engineering, Politehnica University of Timisoara, 2 Piata Victoriei, 300006 Timisoara, Romania; mihaela.ciopec@upt.ro (M.C.); petru.negrea@upt.ro (P.N.); 2 “*Petru Poni*” Institute of Macromolecular Chemistry of Romanian Academy, Iasi, 41A, Gr. Ghica Voda Alley, 700487 Iasi, Romania; biliuta.gabriela@icmpp.ro; 3 Department of Civil Engineering, Indian Institute of Technology Kharagpur, Kharagpur 721302, India; ghangrekar@civil.iitkgp.ac.in

**Keywords:** As(V) removal, viscose fibers, functionalization, adsorption

## Abstract

Exposure to different arsenic concentrations (higher than 10 μg/L), either due to the direct consumption of contaminated drinking water or indirectly by using contaminated food is harmful for human health. Therefore, it is important to remove arsenic from aqueous solutions. Among many arsenic removal technologies, adsorption offers a promising solution with a good efficiency, however the material used as adsorbent play a very vital role. The present investigation evaluated the behavior of two cellulose-based adsorbent materials, i.e., viscose fibers (V) and its TEMPO (2,2,6,6-tetramethylpiperidine-1-oxyl) derivative, obtained by using the well-established TEMPO-mediated protocol (VF). Due to the known arsenic affinity for Fe ions the two materials were later doped with it. This was done after a preliminary functionalization with di-2-ethylhexyl phosphoric acid (DEHPA), to obtain two materials: V-DEHPA-Fe and VF-DEHPA-Fe. Arsenic adsorption is known to be pH dependent (between 6 and 8); therefore, the optimal pH range for As(V) adsorption has been established. In order to evaluate the adsorption mechanism for both the synthesized materials, the influence of contact time, temperature and initial concentration was evaluated. Langmuir, Freundlich and Sips equilibrium isotherm models were used in order to determine the ability of the model to describe As(V) adsorption process. The maximum adsorption capacity of the material V-DEHPA-Fe was 247.5 µg As(V)/g with an As(V) initial concentration of 5 mg/L and for the material VF-DEHPA-Fe it was 171.2 µg As(V)/g with initial concentration of 5 mg/L.

## 1. Introduction

Water is an essential element for life and for all natural processes. The developmental stages of all our economic activities and our existence are totally dependent on this precious resource. It is well known that in developing countries the main source of drinkable water is ground water; hence, contamination of this resource with arsenic represents an acute problem, which needs to be solved to provide safe drinking water to the people. Arsenic is an element which reaches the environment from a variety of natural and anthropogenic sources. Arsenic is a metalloid with properties situated between metals and non-metals, and when As(III) is present in the aquatic environment it is extremely toxic to all life forms [1,2,3,4].

The presence of arsenic compounds in ground water is an acute problem, especially in the Asian region [5], Taiwan, Argentina, West of Bengal, Bangladesh, Mongolia and South and North of America [6,7,8]. Arsenic is considered as a carcinogen, toxic and mutagenic element for humans, hence remediation of arsenic contaminated drinking water poses a global challenge [9,10]. Moreover, arsenic ingestion is associated with skin, bladder, liver and lung cancer [11].

Because of higher toxicity of arsenic, the World Health Organization (WHO) and United States Environmental Protection Agency (US EPA) established a maximum admissible limit of 10 μg As/L. Studies performed during the past decades have proven that the arsenic content in potable waters over this maximum permissible limit has a serious negative impact on human health. Very recently, several measures were taken at international and national levels in order to reduce and even eliminate arsenic from drinking waters [10,12]. The WHO proposed several methods to reduce arsenic levels in drinking-water, such as: discriminate between high- and low-arsenic sources, substitute high-arsenic sources with low-arsenic ones, blending low-arsenic water with high-arsenic water to achieve acceptable levels, and installing arsenic removal systems [13].

Technologies are being developed for removing arsenic from water, such as, coagulation [14,15,16,17,18], oxidation [17,19], reverse osmosis [17,20,21,22], ion exchange [14,15,17,23,24,25,26,27,28,29,30,31,32,33,34,35,36,37,38,39,40,41,42,43,44,45,46,47,48,49], electrocoagulation [50,51,52], adsorption onto fine and coarse iron oxides [53,54] and other materials including iron impregnated activated carbon and natural arsenic adsorbents [17,33,36,37,55,56,57,58,59,60,61,62,63,64,65,66,67,68,69,70]. Taking into account both the arsenic removal efficiency and economic considerations, adsorption represents the most eloquent method, which offers higher design flexibility concomitant with high-quality water treatment. Research performed during the last decade proved that iron oxides increase the affinity of adsorbent materials for arsenic ions. The simultaneous presence of such ions results in high selectivity of adsorbent materials, good chemical stability, low prices, availability, and possibility to produce nano-adsorbents with high specific area. Likewise, the usage of nanoparticles based on ferric oxides to produce the adsorbent materials presents the disadvantage of a low mechanical strength simultaneously with agglomeration trend [71]. However, traditional adsorbents (iron oxide and titanium dioxide based adsorbent) have many limitations (generation of large amount of mud, cannot be reused), which reduce the practical applications and further development of these adsorbents. Thus, the use of natural polymers as an adsorbent material for arsenic removal from water is drawing special attention [72,73,74,75,76,77].

From the environmental point of view, the most common and most cost-effective bio-polymer available from natural sources is cellulose [78,79]. Cellulose, owing to its intrinsic structure, with plenty of OH groups in the anhydroglucose unit, is highly susceptible to chemical transformations, mainly aiming to significantly increase its properties, such as sorption capacity. Cellulose has been proved as an excellent example in terms of versatility for surface modifications [80,81,82]. Introduction of the amino, phosphate and carboxylic terminal groups on cellulose provides efficient resources for precipitating ferric oxide, due to the link established between Fe ions and phosphate groups from di-2-ethylhexyl phosphoric acid (DEHPA) [83]. Moreover, the presence of amino or phosphate fragments influences the synthesis/precipitation of ferric oxides, affecting particle size distribution and colloidal stability into the solution [84].

The main objective of the present study was to prepare and characterize a new adsorbent material for As(V) removal from aqueous solution. In the present study, the 6-carboxy cellulose was synthesized, using viscose fibers as base material by adopting the TEMPO-mediated protocol, and the synthesized product was subsequently used together with the pristine (unoxidized) viscose fibers as supports for further functionalization with pendant phosphor groups, doped with iron (III) ions, and the resulted material was used as an adsorbent for the As(V) ions removal from the arsenic contaminated water. After the preparation of these materials, physico-chemical characterization was performed (Fourier transform infrared spectroscopy, X-ray diffractometry, thermogravimetric analyses), followed by determination of adsorptive properties.

## 2. Materials and Methods

### 2.1. Adsorbent Preparation

Viscose fibers used in this study were donated by Lenzing AG, Lenzing, Austria, having the properties: linear density (dtex) 1.88, average length: 42 mm, mean diameter: 14.6 µm, the degree of polymerization (DPv): 235, molecular mass: 38,500 g/mol, density: 1.5045 g/cm^3^. The 2,2,6,6-Tetramethylpiperidin-1-oxyl (TEMPO, 99% Sigma-Aldrich, St. Louis, MO, USA), sodium bromide (99% Alfa Aesar, Haverhill, MA, USA), and sodium hypochlorite (NaClO, 9% chlorine, SC Chemical Company SRL, Bucharest, Romania) were used as received.

#### 2.1.1. Fiber Preparation

Viscose fibers were washed prior to being used in an aqueous solution of 0.1 M potassium chloride and then acidified using 0.1 M solution of hydrochloric acid. The suspension was stirred for 45 min in order to achieve the complete wetting of the fibers and uniform charge distribution.

#### 2.1.2. Fibers Oxidation

A quantity of 5 g of viscose fibers was suspended in 400 mL deionized water, containing TEMPO (0.0264 g) and sodium bromide (0.26 g). The pH was adjusted to 10 and 21 mL of 9% NaClO solution was added carefully to the cellulose slurry, to start the TEMPO-mediated oxidation. The suspension pH was continuously maintained at 10 using 2 M NaOH solution, under vigorous stirring at room temperature. The oxidation was stopped after 1 h of stirring, by adding ca. 5 mL glycerol. The oxidized cellulose was intensively rinsed with distilled water, and it was recovered by drying under vacuum at 40 °C.

#### 2.1.3. Functionalization of Synthesized Viscose

To improve the adsorbent properties of the viscose fibers (V) and viscose functionalized with –COOH groups (VF) an efficient approach could be their further functionalization using different extractants. In order to obtain functionalization, the oxidized cellulose was dissolved in 0.1 g of extractant di-(2-ethylhexyl) phosphoric acid (DEHPA) (~98.5%, BHD Chemicals Ltd., Poole, UK) in 25 mL ethanol (99.2%, SC PAM Corporation SRL, Bucharest, Romania). The extracted solution is kept in contact with 1 g of synthesized viscose fibers for 24 h. After which the material was separated by filtration and the filtered material was washed with distilled water several times, and then dried in the oven for 24 h at 50 °C. Afterwards the material obtained was loaded with Fe(III) ions using ferric chloride (98.5%, SC Chemical Company SRL, Bucharest, Romania) as iron source. To load the material with Fe^3+^ ions 25 mL of Fe_2_Cl_3_ solution (200 mg/L) was added to the material, left in contact for 24 h, then filtered and dried in an oven for 24 h at 50 °C.

### 2.2. Adsorbent Characterization

#### 2.2.1. Characterization of TEMPO-Mediated Viscose Fibers

##### Fourier Transform Infrared Spectroscopy/Attenuated Total Internal Reflection Spectroscopy (FT-IR/ATR)

FT-IR/ATR experiments were performed on silicon single-crystal parallelepiped internal reflexion elements (IRE) (55 × 5 × 2 mm^3^, 45° incident angle), with a Bruker Vertex 70 instrument (Bruker, Billerica, MA, USA). The FT-IR/ATR spectra were the results of 256 co-added scans with a resolution of 4 cm^−1^.

##### X-ray Diffractometry

The pristine and TEMPO-oxidized viscose fibers were converted to pellets using a disk apparatus (Bruker, Billerica, MA, USA) and subjected to X-ray diffraction analysis in the range of 5° to 35°, 2*θ* diffraction angle. The determinations were performed in Bragg–Bretagne geometry employing a D8 Advance Bruker X-ray diffractometer (Bruker, Billerica, MA, USA) using Cu Kα radiation (α = 0.1548 nm) at 30 KV and 36 mA. The diffractometer used a step width of 0.02° and a time constant of 0.4 s per step.

The degrees of crystallinity (DC) of the neat and oxidized viscose samples were determined from the area of the amorphous and crystalline regions by using DiffracPlus Bruker TOPAS software (version 7), using formula: DC = 100 × crystalline area/(crystalline area + amorphous area). Lorentz-polarization corrections and pseudo-Voight functions were applied in the peaks profile fitting.

##### Thermogravimetric Analyses

Samples of viscose fibers, before and after oxidation (~10 mg) were deposited into Al_2_O_3_ crucibles followed by heating under nitrogen, from 30 to 700 °C, with a 10 °C min^−1^ heating rate, using a thermal analyzer STA 449 F1 Jupiter device (Netzsch, Selb, Germany). Thermogravimetric (TG) and derivative thermogravimetric (DTG) curves, recorded with a ± 0.5 °C precision were processed employing a Netzsch Proteus analysis software (version 6.1).

#### 2.2.2. Characterization of the Adsorbent

The adsorbent obtained were characterized by recording the SEM micrographs, energy dispersive X-ray spectra (EDX) and Fourier transformed infrared spectra (FT-IR). SEM images and EDX spectra were recorded using a scanning electron microscope FEI Quanta FEG 250 (FEI, Hillsboro, OR, USA) and the FT-IR spectra were recorded by using a Bruker Platinum ATR-QL Diamond infra-red spectrometer (Bruker, Billerica, MA, USA).

### 2.3. Bach Adsorption Experiments

In order to determine As(V) adsorption mechanism onto the synthesized materials firstly the effect of different physical-chemical parameters, such as solution pH, contact time, As(V) initial concentration, and temperature, onto the materials adsorption capacity, was evaluated. Later kinetic, equilibrium and thermodynamic studies were performed in order to determine the performance of viscose materials functionalized with COOH, P and Fe(III) groups for As(V) removal from aqueous solutions.

One of the significant variables which offers an important effect onto the affinity of adsorbent materials for a specific ion is the solution pH. Its influence is related with the form of metallic ions present into the solution as well as the functional groups of the extractant used. In order to determine the optimum pH for the As adsorption onto the functionalized viscose materials, the pH values were varied in range of 2–12 for an initial As concentration of 1 mg/L and a contact time of 60 min and temperature of 298 K was maintained. The pH values were corrected by using the HCl solution with concentration between 0.01 and 1 M, and NaOH solutions with concentrations between 0.05 and 1 M.

In order to study the influence of contact time onto the adsorption capacity of synthesized adsorbent material, 25 mL As(V) ion solution with an initial concentration of 1 mg/L was added in the synthesized material (0.1 g). Samples were stirred for time intervals of 5, 15, 30, 45, 60, 90, 120, 180 and 240 min at different temperatures (273, 308 and 318 K). The effect of the initial concentration onto the adsorption capacity and the equilibrium concentration were established by using As(V) solution with different initial concentrations (0.2, 0.4, 0.6, 0.8, 1, 2, 3, 5, 6, 7 mg/L), which were obtained by dilution from a stock solution containing 1000 mg As(V)/L.

All adsorption processes were carried out in a static regime using a Julabo shaker at 200 rotations per minute. Analysis of residual As(V) concentration in the solutions after the adsorption process was carried out using inductively coupled plasma mass spectrometry Aurora M90 from Bruker (Billerica, MA, USA). Adsorption capacity q (μg/g) of used adsorbent material was estimated using Equation (1):

(1) $$q=\frac{\left(C_{0}-{\;C}_{f}\right)V}{m}$$

where, C_0_—initial concentration of As(V) of the solution, (μg/L); C_f_—residual concentration of As(V) of the solution, (μg/L); V—volume of solution (L); m—mass of adsorbent material, (g).

## 3. Results and Discussions

### 3.1. Characterization of Synthesized Viscose

**Figure 1:**
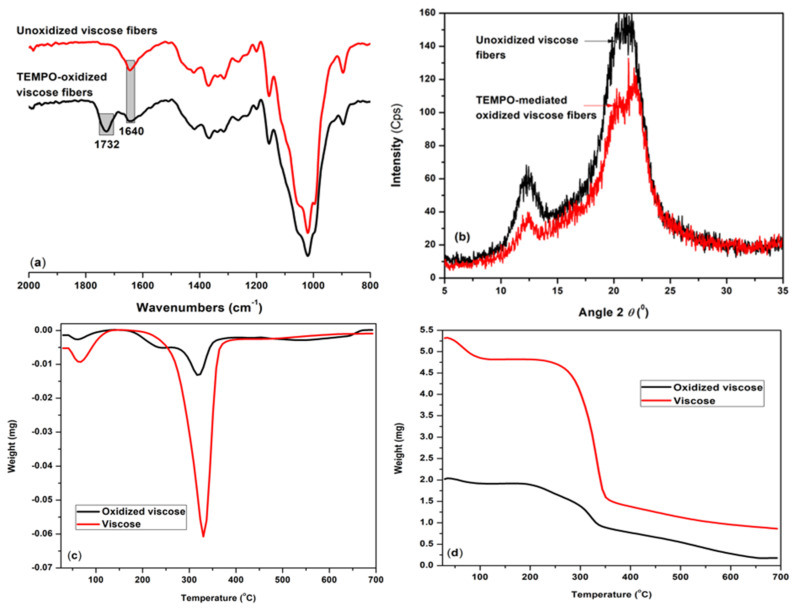
The FTIR-ATR spectra (**a**), X-ray diffraction patterns (**b**), derivative thermogravimetric (DTG) (**c**) and therm-gravimetric (TG) (**d**) analyses of unoxidized (V) and TEMPO oxidized (VF) samples.

Infrared spectroscopy can be used as a powerful technique to check the successful conversion of the OH groups into COOH groups during cellulose oxidation [85,86]. The TEMPO-mediated oxidation of viscose fibers allows the formation of significant amounts of 6-carboxy cellulose, with increasing amount from 6 mmol/kg in the unoxidized sample to 280 mmol/kg in the oxidized sample, as determined by using potentiometric titration [81,87]. The main adsorption bands related to hydrogen bond stretching vibration of the OH groups at 3345 cm^−1^ and to stretching vibrations of CH at 2905 cm^−1^ are nearly unchanged after oxidation. Figure 1a indicates the fragments of the FTIR spectra of the most visible transformations. These are mostly related with the apparition of a characteristic adsorption for carboxylate groups at 1732 cm^−1^ assigned with COO^−^ groups in their acidic forms [85,86]. The crystallinity pattern of the oxidized sample does not essentially differ from that of the pristine viscose, suggesting that the oxidation treatment did not affect the crystalline structure of the cellulose, Figure 1b. The measured crystallinity increased after oxidation from about 25% in the original sample to about 28%, this increase in the crystallinity might be due to removal of small amorphous fragments, as water soluble by-product, favored by the alkaline media.

As seen in Figure 1c,d, thermogravimetric curves revealed the main changes of the cellulosic materials during heating. The total loss of free water in viscose fibers was observed around 100 °C, whereas for the TEMPO-oxidized viscose fibers, the temperature is slightly lower, around 97 °C. Moreover, the unoxidized fibers exhibited higher loss of free water (~9.5%) as compared to the oxidized fibers (~6%). Between 280 and 350 °C, a second stage of sample degradation for viscose can be seen, corresponding to degradation of hemicelluloses, which is more sensitive to temperature fraction than cellulose itself because of its amorphous character. The introduction of carboxyl groups on the viscose surface, significantly affected the thermal stability of the resulted sample, with first degradation occurring at 80 °C which was before than in the case of neat viscose. Two-stage decomposition in the DTG curve is observed in the case of oxidized viscose, with a degradation temperature range of 202–335 °C (reaching a leading peak at 319 °C) and the second stage at higher temperatures, with a leading peak at 542 °C. This degradation step at high temperature is caused as previously reported due to the very strong intermolecular-hydrogen bonding occurring in the crystalline areas of cellulose [88].

### 3.2. Characterized of V-DEHPA-Fe and VF-DEHPA-Fe Materials

#### 3.2.1. X-ray Energy Dispersive Spectroscopy

**Figure 2:**
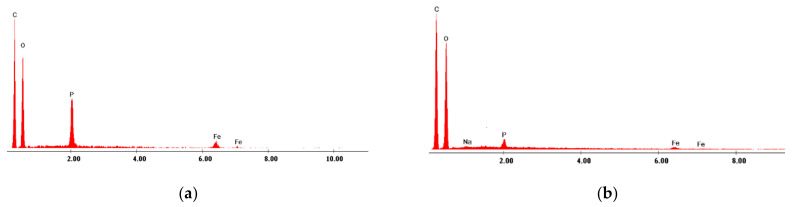
EDX spectrum of materials (**a**) V-DEHPA-Fe and (**b**) VF-DEHPA-Fe.

**Figure 3:**
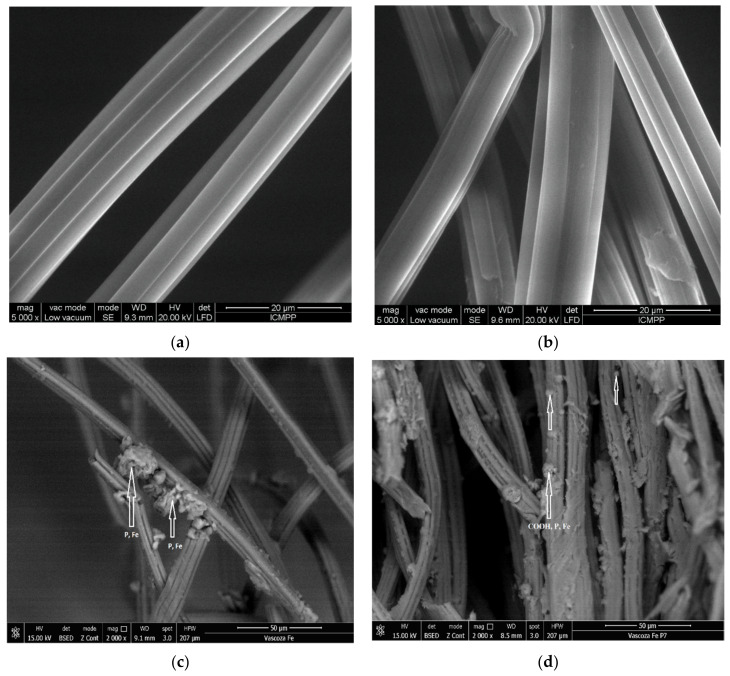
SEM imagines of unoxidized (V) (**a**), TEMPO-oxidized (VF) and the resulted materials, (**b**) V-DEHPA-Fe (**c**) and VF-DEHPA-Fe (**d**), viscose fibers.

In order to prove that viscose materials used as adsorbent were functionalized with di-2-ethylhexyl phosphoric acid, the EDX spectra (Figure 2) and the SEM pictures (Figure 3) were obtained. By analyzing the EDX spectra (Figure 2) the presence of peaks of C and O can be seen, which are associated with the chemical structure of the viscose. Likewise, the presence of P and Fe peaks, which are associated with the presence of the used extractant and Fe ions onto the viscose surface, indicated that the used material was functionalized and doped with Fe ions. By comparing the two spectra presented in Figure 2, it can be inferred that the P and Fe peaks recorded in the case of V-DEHPA-Fe adsorbent are higher than the peaks observed in the case of VF-DEHPA-Fe adsorbent. This difference can be explained taking into account that the VF used as support for functionalization with DEHPA and doped with Fe ions with high content of COOH groups, that can block the active sites of viscose preventing in this way the attachment of P and Fe onto the surface.

SEM pictures (Figure 3), reveal the main changes that occurred in the morphology of the viscose fibers. The fibrillar structure of the viscose fibers is maintained after the TEMPO-mediated oxidation, which proved the “surface” character of the protocol, Figure 3a,b. On the other hands, the presence of some micro-granules fixed onto the surface of viscose fibers after extractant and Fe ions incorporation can be observed, Figure 3c,d. The presence of such micro-granules can be attributed to the successful immobilization of the phosphorous groups and Fe atoms on the fibers surface, as a consequence of material functionalization.

#### 3.2.2. FTIR Infrared Spectroscopy Analysis with Fourier Transformed

**Figure 4:**
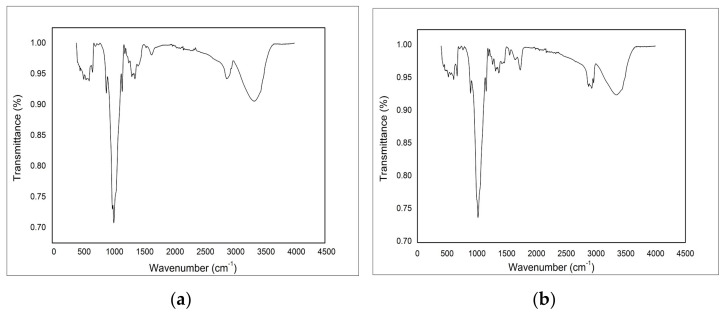
FTIR spectra of (**a**) V-DEHPA-Fe and (**b**) VF-DEHPA-Fe.

In order to highlight the presence of polymer (viscose—V, and viscose synthesized with carboxyl groups) specific groups, extractant (DEHPA) and Fe were recorded the FT-IR spectra as depicted in Figure 4. From FT-IR spectra recorded for the two produced adsorbent materials the presence of the bands characteristic of viscose can be observed. At wavelength of 2900 cm^−1^ a band specific to the stretching vibrations of the C–H bonds was observed [89] and around the wavelengths of 1600 and 1418 cm^−1^, two peaks associated with strong symmetric/asymmetric vibrations of C=O bonds were observed [90]. At the same time, in the case of chemically modified viscose (VF) (Figure 3b), a specific vibration was observed at 2900 cm^−1^, which can be associated with the transformation of the –CH_2_–OH bond in the –COOH bond [90].

Additionally, vibrations specific to P–OH bonds at 2340 and 1654 cm^−1^ were observed. The peak observed at 1230 cm^−1^ is specific for the stretching vibrations of P=O bonds and peak observed at 1155 cm^−1^ is specific for the vibrations of P–O–C bonds, which occurred due to the presence of di-(2-diethyl-hexyl) phosphoric acid-DEHPA in the viscose matrix used as extractant for functionalization. Specific vibrations due to the presence of Fe(III) on the viscose surface can be observed at 1037 cm^−1^, which are characteristic to the symmetric stretching vibrations of the Fe–OH bond [91].

#### 3.2.3. Effect of pH

**Figure 5:**
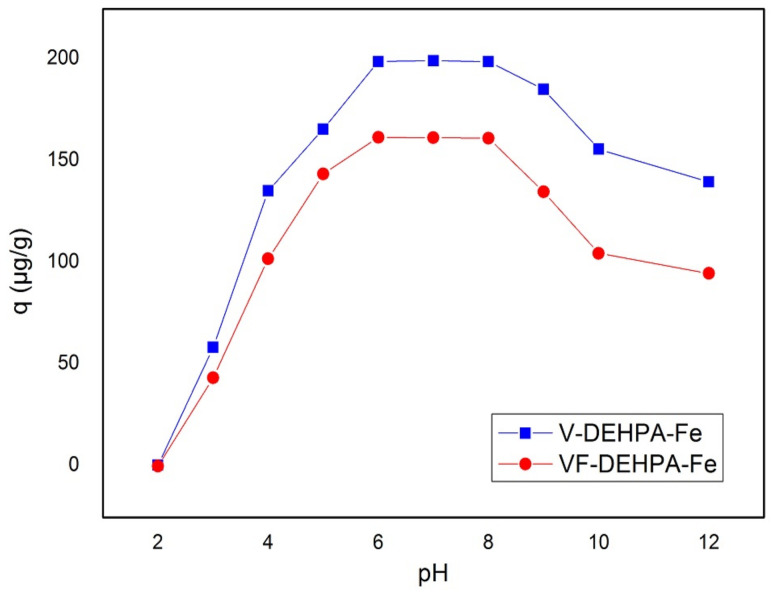
Effect of pH on adsorption capacity of As(V) on functionalized materials.

Effect of pH on the adsorption capacity of As(V) on V-DEHPA-Fe and VF-DEHPA-Fe materials, at an initial concentration of As(V) C_0_ = 1 mg/L at contact time of 60 min and temperature of 298 K was evaluated (Figure 5). The studies were carried out in the pH range of 2–12. From the performance results it can be inferred that for both adsorbent materials, viz. V-DEHPA-Fe and VF-DEHPA-Fe, the adsorption capacity increases with the increase in pH up to 6.0 and beyond 8.0 the adsorption capacity was observed to decrease indicating optimum pH in the range of 6.0–8.0.

Understanding the dependence of As(V) adsorption capacity/pH is complex to discuss because the different As(V) species exist in water at different pH [92]:

(2) $$H_{2}{\mathrm{AsO}}_{4}\;↔{\;H}^{+}+{\;H}_{2}{\mathrm{AsO}}_{4}^{-}{\;\mathrm{pK}}_{1}=2.3$$

(3) $$H_{2}{\mathrm{AsO}}_{4}^{-}\;↔{\;H}^{+}+{\;H}_{2}{\mathrm{AsO}}_{4}^{2-}{\;\mathrm{pK}}_{2}=6.8$$

(4) $$H_{2}{\mathrm{AsO}}_{4}^{2-}\;↔{\;H}^{+}+{\;\mathrm{AsO}}_{4}^{3-}{\;\mathrm{pK}}_{1}=11.6$$

From Equations (2)–(4), it can be observed that in the acidic condition the dominant As(V) species is $H_{2}{\mathrm{AsO}}_{4}^{-}$, having a stronger adsorption ability than water. In the case of neutral and week basic conditions, the predominant species is $H_{2}{\mathrm{AsO}}_{4}^{2-}$, and ${\mathrm{AsO}}_{4}^{3-}$ will be predominant at high pH conditions [92]. The decrease in the maximum adsorption capacity at pH above 8 can be associated with the competitive adsorption between As(V) species and OH^−^ ions.

In the literature it is known that in aqueous solutions As(V) exists in four species forms: H_3_AsO_4_, H_2_AsO_4_^−^, HAsO_4_^2−^, AsO_4_^3−^ and in the pH range of 6 to 8, where the maximum adsorption capacities was obtained, the As(V) can be found as H_2_AsO_4_^−^ and/or HAsO_4_^2−^ [36]. Hence, further adsorption studies were carried out in the pH range of 6–8, the interval in which the highest adsorption capacity values were obtained.

#### 3.2.4. Effect of Contact Time and Temperature and Adsorption Kinetics/Thermodynamics

**Figure 6:**
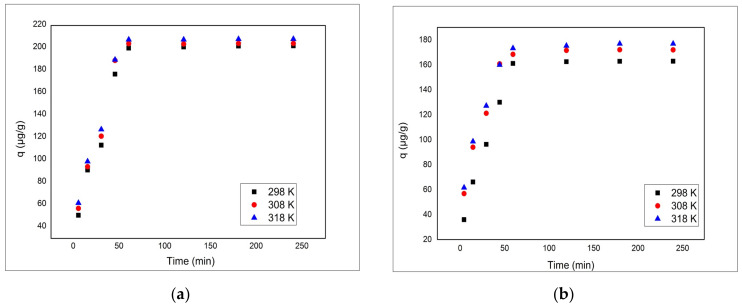
Effect of contact time and temperature on adsorption capacity of materials: (**a**) V-DEHPA-Fe and (**b**) VF-DEHPA-Fe (m = 0.1 g, v = 25 mL, C_0_ = 1 mg L^−1^ As(V), pH = 7).

The effect of contact time on the adsorption of As(V) onto V-DEHPA-Fe and VF-DEHPA-Fe materials at three different operating temperatures is presented in Figure 6a,b, indicating that for both materials the process takes place in three steps. In the first step, up to 60 min contact time, adsorption capacity sharply increased due to a very fast As(V) uptake. This step is attributed to bulk diffusion in which the most readily available adsorbing sites on the adsorbent surface are used very fast for both the adsorbent materials used. In the second step, up to 120 min for both materials (V-DEHPA-Fe and VF-DEHPA-Fe) uptake gradually decreased with time until it reached equilibrium. This transition phase is attributed to intra-particle diffusion, meaning the diffusion of adsorbate from the superficial film into the macro-pores of the adsorbent. The third step represents the equilibrium stage in which the removal of the adsorbate becomes almost insignificant, due to the exhaustion of adsorption active sites. For equilibrium studies, a contact time of 120 min was chosen for both materials. Additionally, the adsorption capacity of As(V) slightly increased with temperature increased from 298 to 318 K, indicating that the temperature has a little influence on the adsorption of As(V) onto the adsorbent materials used.

The kinetics study of As(V) adsorption onto the synthesized materials was performed in order to find the equilibrium time for the adsorption process and to elucidate the mechanism which control As(V) adsorption. In order to elucidate the adsorption mechanism, the experimental results were modeled using the pseudo-first order (Lagergren) and the pseudo-second order (Ho and McKay) kinetic models. The kinetics of the adsorption process of As(V) on two materials viz, V-DEHPA-Fe and VF-DEHPA-Fe was studied. Experiments were carried out with a view to find out the equilibrium time of adsorption and the mechanism of adsorption. The pseudo-first-order kinetic equation can be expressed as Equation (5).

(5) $$\frac{{\mathrm{dq}}_{t}}{\mathrm{dt}}={\;k}_{1}\left(q_{e}-{\;q}_{t}\right)$$

where, q_e_ and q_t_ are the adsorbed amounts of As(V) per unit mass of material at equilibrium and time t, respectively (µg/g) and k_1_ is the pseudo-first-order rate constant (min^−1^).

The q_t_ at different time t can be determined by using the following pseudo-first-order kinetic equation obtained after integration of Equation (5) to get Equation (6).

ln (q_e_ − q_t_) = ln q_e_ − k_1_t (6)

The pseudo-second-order kinetic model is described by the Equation (7).

(7) $$\frac{{\mathrm{dq}}_{t}}{\mathrm{dt}}={\;k}_{2}{\left(q_{e}-{\;q}_{t}\right)}^{2}$$

where, k_2_ is the pseudo-second-order rate constant (min^−1^ (µg/g)^−1^). The linearized form of the Equation (7) could be expressed as Equation (8).

(8) $$\frac{t}{q_{t}}=\frac{1}{k_{2}q_{e}^{2}}+\frac{t}{q_{e}}$$

**Figure 7:**
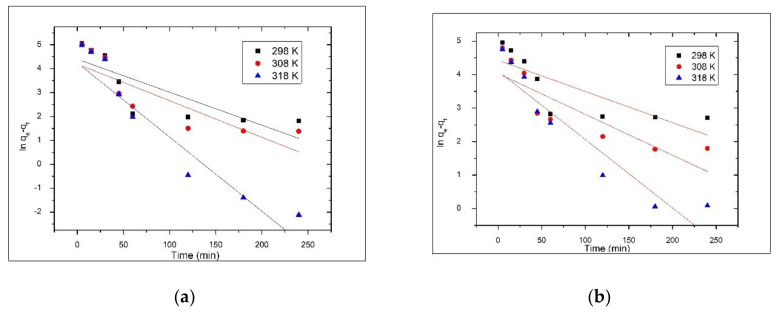
Pseudo-first-order kinetic model for As(V) adsorption onto functionalized materials (**a**) V-DEHPA-Fe and (**b**) VF-DEHPA-Fe, at different temperatures.

**Figure 8:**
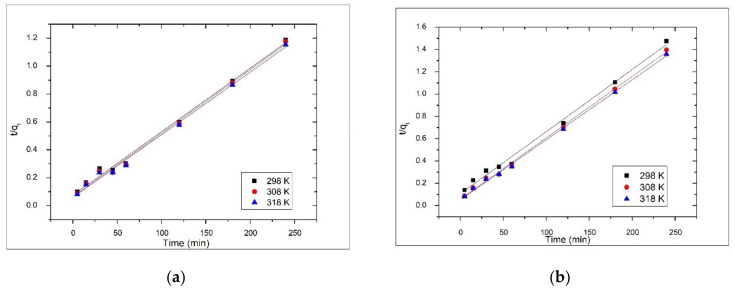
Pseudo-second-order kinetic model for As(V) adsorption onto functionalized materials (**a**) V-DEHPA-Fe and (**b**) VF-DEHPA-Fe, at different temperatures.

The rate constant (k_1_) and the equilibrium adsorption capacity (q_e_) for pseudo-first-order kinetic model were determined from the linear dependence of ln(q_e_ − q_t_) versus t (Figure 7) and the second-order rate constant (k_2_) and the equilibrium adsorption capacity (q_e_) for pseudo-second-order kinetic model were determined from the linear plot of t/q_t_ versus t (Figure 8) for all the temperatures at which adsorption was performed. The values of the constants, together with the regression coefficients (R^2^) and the estimated errors obtained for both materials are presented in Table 1 and Table 2. Depending on the values of rate constants and regression coefficients (R^2^) obtained, the kinetic model that best describes the adsorption process was established.

It was observed that the correlation coefficients for the pseudo-first-order model are much lower than the values of the correlation coefficients obtained for the pseudo-second-order model, which are close to 1 for both materials. Additionally, for the pseudo-first-order model, there was a large difference between the values of q_e_ as determined experimentally (q_e,exp_) and those calculated from the kinetic plots (q_e,calc_). In the case of the pseudo-second-order model, the theoretically predicted equilibrium adsorption capacities are close to those experimentally determined, at all temperatures. At the same time, in the case of the pseudo-first-order model, the values of the estimated error χ^2^ were very high, which means that the experimental data do not fit the kinetic model. This shows that the kinetics of the As(V) ion removal process by adsorption on the V-DEHPA-Fe and VF-DEHPA-Fe materials is well described by the pseudo-second-order model. For both materials, the rate constant (k_2_) increased with temperature, this demonstrates that the adsorption process of As(V) on the synthesized materials is an endothermic process.

**Figure 9:**
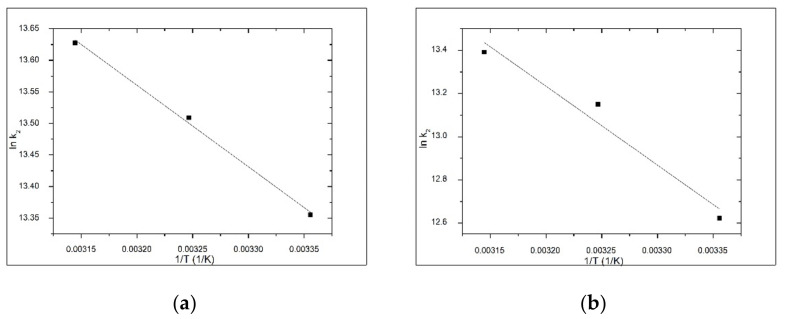
Arrhenius plot of the adsorption of As(V) onto functionalized materials (**a**) V-DEHPA-Fe and (**b**) VF-DEHPA-Fe.

For adsorption As(V) on V-DEHPA-Fe and VF-DEHPA-Fe materials, activation energy (E_a_) was calculated using the Arrhenius equation and the rate constant (k_2_) obtained from the pseudo-second order kinetic model. The activation energy associated with the As(V) adsorption onto these materials was calculated from the linear dependence of ln k_2_ versus 1/T (Figure 9).

Based on the experimental data obtained, the value of activation energy for V-DEHPA-Fe was found to be 10.74 kJ mol^−1^ with a coefficient of determination of R^2^ = 0.9967. Similarly, for VF-DEHPA-Fe activation energy of 30.53 kJ mol^−1^ and coefficient of determination of R^2^ = 0.8424 suggest that the adsorption process of As(V) on the materials evaluated is a chemical adsorption, because the activation energy has value higher than 8 kJ mol^−1^ [93,94]. Thermodynamic studies were conducted in the temperature range 298 to 318 K. These studies provide information about the energy changes associated with the adsorption process, confirming if the process is spontaneous or not. Specific thermodynamic parameters free energy (ΔG^0^), free enthalpy (ΔH^0^) and free entropy (ΔS^0^) were calculated by using relations as provided in Equation (9) through (11).

ΔG^0^ = −RTlnK_c_ (9)

(10) $$\mathrm{where},\;K_{d}=\frac{C_{\mathrm{Ae}}}{C_{e}},$$

(11) $$\mathrm{and}\;\log K_{d}=\frac{\Delta S^{0}}{2.3\;R}-\frac{\Delta H^{0}}{2.303\;\mathrm{RT}}$$

where: R is the gas constant, K_d_ is the equilibrium constant, T is the temperature (K), C_Ae_ is the equilibrium concentration As(V) on adsorbent (μg/L), and C_e_ is the equilibrium concentration of As(V) in the solution (μg/L).

**Figure 10:**
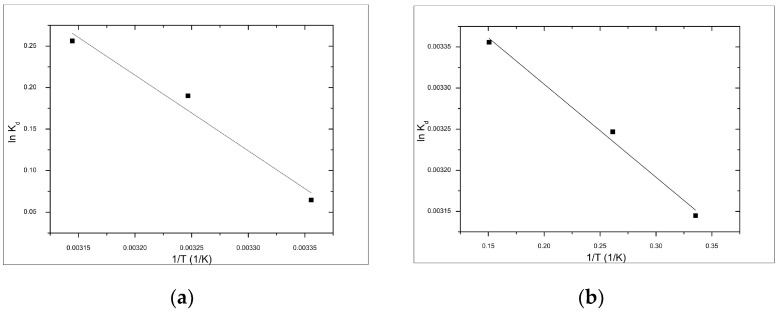
Van’t Hoff plots for the adsorption of As(V) onto functionalized materials (**a**) V-DEHPA-Fe and (**b**) VF-DEHPA-Fe.

In order to evaluate the thermodynamic parameters (presented in Table 3) the linear dependences of ln k_d_ versus 1/T (1/K) for both adsorbents were plotted as shown in Figure 10.

The negative values obtained for free Gibbs energy (ΔG^0^) suggest that the adsorption process of As(V) on functionalized materials is a spontaneous and natural process. The value of free Gibbs energy become more negative with the temperature increase, which can be associated to effective growth of the contact surface between the adsorbent material and As(V) ions. Free enthalpy of less than 80 kJ/mol indicated that the studied adsorption process is a physical-chemical one. This suggests that the optimum adsorption process occurs at higher temperatures. From data presented in Table 3, it can be observed that the ΔH^0^ have positive values for both studied adsorbent materials, which implies the endothermic nature of As(V) adsorption. The positive value of entropy (ΔS^0^) suggests that adsorption increases in randomness at the material/solution interface, and the degree of clutter of the particles increases with increasing temperature, which can be attributed to the material surface changes [83]. Regarding the adsorption mechanism, it can be considered that the Fe will be bonded through coordinative bonds with phosphate groups from DEHPA molecules, and further As(V) ions will be retained through ionic bonds by the formed complex [83].

### 3.3. The Effect of Initial As(V) Concentration and Adsorption Isotherms

**Figure 11:**
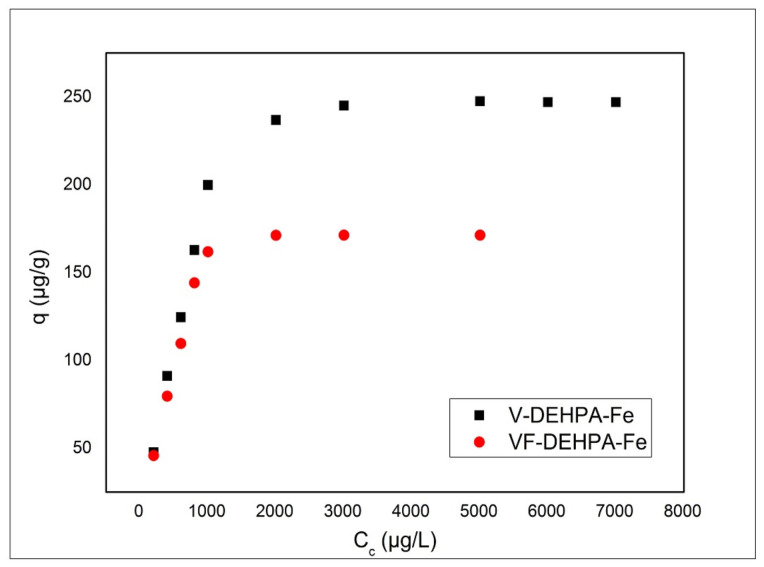
Effect of initial concentration of As(V) on the adsorption process.

Figure 11 presents the effect of initial As(V) concentration on the adsorption process. From the image depicted in Figure 11 it can be observed that the adsorption capacity increases with increasing of the initial concentration of As(V) for both materials until it reaches a constant value, known as the equilibrium concentration. The maximum adsorption capacity represents an important parameter for designing an adsorption system. Thus, the maximum adsorption capacity of the material V-DEHPA-Fe was 247.5 µg As(V)/g for an initial As(V) concentration of 5 mg As(V)/L and for the material VF-DEHPA-Fe it was 171.2 µg As(V)/g for an initial As(V) concentration of 5 mg As(V)/L.

From the experimentally obtained data it is observed that the material which was synthesized with carboxyl groups (VF-DEHPA-Fe) has a lower adsorption capacity than the V-DEHPA-Fe material, which can be due to the –COOH groups that occupy a part of the viscose surface, preventing in this way the binding of phosphorus and Fe(IIII) groups. The adsorption isotherms determine the relationship between the concentration and the amount of metal ion adsorbed per unit mass of adsorbent at constant temperature. The Langmuir, Freundlich and Sips isotherms have been used to model experimental data in order to establish the adsorption mechanism and the maximum adsorption capacity of these two materials used [95,96,97].

Non-linear expression of the Langmuir isotherm equation [98] can be expressed as follows (Equation (12)):

(12) $$q_{e}=\frac{q_{\max}K_{L}C_{f}}{1+K_{L}C_{f}}$$

where, q_e_—the maximum absorption capacity (µg/g); C_f_ the equilibrium concentration or final concentration of As(V) in solution (µg/L); q_max_—Langmuir maximum adsorption capacity (µg/g); K_L_—Langmuir constant.

Freundlich isotherm can be applied to heterogeneous adsorption surfaces [96]. Non-linear form of the Freundlich isotherm is expressed as Equation (13) [99]:

(13) $$q_{e}=K_{F}C_{f}^{1/n_{f}}$$

where, q_e_—the maximum absorption capacity (µg/g); C_f_—the equilibrium concentration or final concentration of As(V) in solution (µg/g); K_F_ și n_f_—the characteristic constants that can be related to the relative adsorption capacity of the adsorbent and the intensity of adsorption. The Sips isotherm represents a combination between Freundlich and Langmuir isotherms (Equation (14)) [100].

(14) $$q_{e}=\frac{q_{s}K_{S}C_{e}^{1/n_{S}}}{1+K_{S}C_{e}^{1/n_{S}}}$$

where, q_S_—the maximum absorption capacity (µg/g); K_S_—constant related to the adsorption capacity of the adsorbent; and n_S_—the heterogeneity factor.

**Figure 12:**
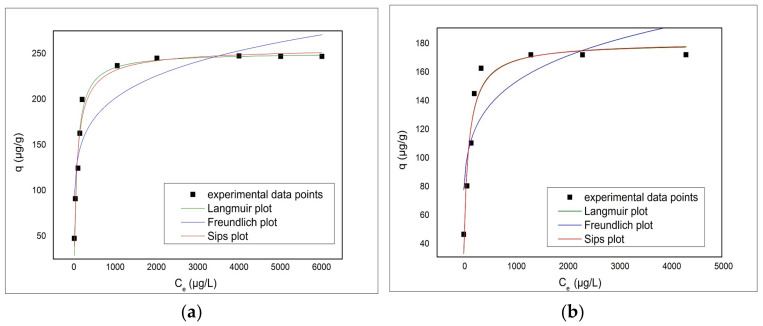
Adsorption isotherms of As(V) onto functionalized materials (**a**) V-DEHPA-Fe and (**b**) VF-DEHPA-Fe.

Figure 12 presents the Freundlich, Langmuir and Sips adsorption isotherms obtained by modeling the experimental data with these isotherm models. Based on isotherm obtained the parameters associated with As(V) adsorption were determined and presented in Table 4.

From Table 4 it is observed that the adsorption process of As(V) on the V-DEHPA-Fe material is better described by the Sips model, with the regression coefficient R^2^ = 0.9747. It is also observed that the maximum adsorption capacity theoretically obtained by modeling the experimental data with Sips isotherm is q_S_ = 257.3 µg/g, a value very close to the experimental data value q_m,exp_ = 247.5 µg/g. This value represents a confirmation that the Sips model better describes the adsorption process of As(V) on this material. In the case of adsorption As(V) on VF-DEHPA-Fe material, the Langmuir isotherm best fits the experimental data. The maximum adsorption capacity obtained theoretically for the VF-DEHPA-Fe material was q_L_ = 179.7 μg/g. This value was close to the observed maximum experimental adsorption capacity q_m,exp_ = 171.2 μg/g. The coefficient of determination is R^2^ = 0.9398. The linear representation of the Freundlich isotherm for both materials has very low correlation coefficients suggesting that this isotherm cannot describe the As adsorption onto the studied materials.

Additionally, a comparison of different adsorbent materials and their maximum adsorption capacities obtained for As removal are presented in Table 5. From data presented in Table 5 it can be observed that by using SiO_2_ and MgSiO_3_, the lowest adsorption capacities for As(V) removal were obtained. Adsorption capacities were increased by functionalization of polymeric materials with different extractants. The highest adsorption capacity (247.5 μg/g) has been obtained for functionalized viscose fibers.

## 4. Conclusions

The results reported in this study have shown that the new adsorbent materials obtained from functionalization of synthesized viscose with DEHPA and Fe(III) ions are effective for increasing As(V) removal efficiency from aqueous solutions. The maximum adsorption capacity of the material V-DEHPA-Fe was 247.5 µg As(V)/g with an As(V) initial concentration of 5 mg/L, and for the material VF-DEHPA-Fe it was 171.2 µg As(V)/g with an As(V) initial concentration of 2 mg/L. The required contact time was observed to be 120 min. The mechanism describing the adsorption process of As(V) onto the functionalized materials was established via kinetic, thermodynamic and equilibrium studies. The results indicate that the adsorption process takes place at the surface of the material, being a spontaneous, endothermic process, and the nature of the interactions between the metal ions and the surface of the material may be chemical bonds. The adsorption kinetics were better described by the pseudo-second-order kinetic model for both materials, and the adsorption isotherms that best represent the process are the Sips isotherm for V-DEHPA-Fe and the Langmuir isotherm for VF-DEHPA-Fe.

## Figures and Tables

**Table 1:** Kinetic parameters for the adsorption of As(V) onto material V-DEHPA-Fe.

Temp, (K)	q_e,exp_, µg g^−1^	Pseudo-First Order Kinetic Model				Pseudo-Second Order Kinetic Model			
		q_e,calc_, µg g^−1^	k_s1_, min^−1^	R^2^	χ^2^	q_e,calc_, µg g^−1^	k_s2_, min^−1^ (µg/g)^−1^	R^2^	χ^2^
298	199.8	80.88	0.0138	0.6252	0.46	217.4	63.1·10^4^	0.9920	1.5·10^−4^
308	203.8	65.89	0.0153	0.5624	0.57	217.4	73.6·10^4^	0.9928	1.4·10^−4^
318	207.4	69.48	0.0310	0.7424	0.79	222.2	82.8·10^4^	0.9941	1.3·10^−4^

**Table 2:** Kinetic parameters for the adsorption of As(V) onto material VF-DEHPA-Fe.

Temp, (K)	q_e,exp_, µ/g^−1^	Pseudo-First Order Kinetic Model				Pseudo-Second Order Kinetic Model			
		q_e,calc_, µg g^−1^	k_s1_, min^−1^	R^2^	χ^2^	q_e,calc_, µg g^−1^	k_s2_, min^−1^ (µg/g)^−1^	R^2^	χ^2^
298	161.1	83.82	0.0093	0.6015	0.32	178.6	30.3·10^4^	0.9927	1.7·10^−4^
308	168.4	56.49	0.0122	0.6206	0.41	181.8	60.3·10^4^	0.9975	1.0·10^−4^
318	173.3	60.77	0.0204	0.8041	0.44	185.2	65.4·10^4^	0.9982	8.5·10^−5^

**Table 3:** Thermodynamic parameters for the adsorption of As(V) onto the functionalized materials.

Temperature, K	V-DEHPA-Fe				V-DEHPA-Fe			
	K_d_, L g^−1^	ΔG^0^, kJ mol^−1^	ΔH^0^, kJ mol^−1^	ΔS^0^, kJ (mol K)^−1^	K_d_, L g^−1^	ΔG^0^, kJ mol^−1^	ΔH^0^, kJ mol^−1^	ΔS^0^, kJ (mol K)^−1^
298	1.06	−0.18	7.57	26.01	1.16	−0.38	7.28	25.74
308	1.21	−0.44			1.29	−0.64		
318	1.29	−0.71			1.39	−0.90		

**Table 4:** Parameters of Freundlich, Langmuir and Sips isotherms for As(V) adsorption onto functionalized materials.

Adsorption Isotherms	Parameters	V-DEHPA-Fe	VF-DEHPA-Fe
	Experimental Values		
	q_m,exp_ (µg g^−1^)	247.5	171.2
**Isotherm Models Values**			
Langmuir	q_L_ (µg g^−1^)	251.3	179.7
	K_L_ (L mg^−1^)	0.014	0.013
	R^2^	0.9728	0.9398
Freundlich	K_F_ (L mg^−1^)	64.13	48.23
	1/n_F_	0.165	0.165
	R^2^	0.8317	0.7229
Sips	q_S_ (µg/g)	257.3	180.4
	K_S_	0.028	0.015
	1/n_S_	0.164	0.029
	R^2^	0.9747	0.9280

**Table 5:** Comparison between adsorption capacities obtained for different adsorbent materials.

Material	Adsorption Capacity q (μg/g)	Reference
Fe-XAD7-DEHPA	15.7	[101]
XAD8-DEHPA-Fe	13	[91]
IR-120 (Na)-DEHPA-Fe	21.8	[101]
XAD-7-DEHPA-TOPO-Fe	35.5	[102]
XAD-7-DEHPA-TPPO-Fe	29.8	[102]
Dibenzo-18-crown-6-Fe	30	[83]
SiO_2_	21.5	[103]
MgSiO_3_	2.75	[103]
V-DEHPA-Fe	247.5	Present paper
VF-DEHPA-Fe	171.2	Present paper

## Data Availability

Data sharing is not applicable to this article.
